# Lessons Learned: Asphyxiation Hazard Associated with Dry Ice

**DOI:** 10.1021/acs.chas.3c00027

**Published:** 2023-05-08

**Authors:** Bonnie J. Park, Christopher D. Vanderwal

**Affiliations:** † Department of Chemistry, 1102 Natural Sciences II, University of California, Irvine, California 92697-2025, United States; ‡ Department of Pharmaceutical Sciences, 101 Theory #100, University of California, Irvine, California 92617, United States

**Keywords:** dry ice, laboratory safety, asphyxiation hazard, lessons learned, CO_2_ poisoning

## Abstract

Dry ice is widely used in the chemistry research settings as an excellent coolant. Herein, we report a case study of a graduate student researcher who lost consciousness while retrieving 180 lbs of dry ice from a deep dry ice container. We share the details of the incident and the lessons learned from it to promote safer handling of dry ice in these situations.

## Introduction

Dry ice is the solid form of carbon dioxide (CAS No. 124-38-9) that sublimes into gaseous carbon dioxide, which is heavier than air, at −78.5 °C at atmospheric pressure.^1^ It is used to preserve foods, flash-freeze tissues, transport temperature-sensitive products, and provide dramatic visual effects. In the chemistry laboratory setting, dry ice is used in combination with organic solvents to generate cooling baths that can maintain reaction mixtures (within flasks partially submerged in the cooling bath) at cryogenic temperatures.^1^ Herein, we describe an asphyxiation incident during the retrieval of a large quantity of dry ice and share the lessons learned. We have included a brief overview of safety incidents with dry ice, as well as the physical and mental health effects from such events.

## What happened?

Prior to the COVID-19 pandemic, dry ice was delivered from the supply company to individual research laboratories in the Department of Chemistry at UC Irvine. However, since COVID-19, the lab-specific delivery service stopped, and the laboratories are now largely dependent upon the purchase of dry ice from the School of Physical Science Stores (PS Stores). Each dry ice pack thus supplied weighs 30–50 lb (14–23 kg) and is wrapped in brown paper for handling.

On Monday, May 30th, 2022 (Memorial Day), around 8 a.m., a graduate student researcher visited PS Stores with a cart to retrieve dry ice for their research group. The general protocol for the lab was that ∼200 lb (90.7 kg) of dry ice would be purchased from the store and brought up to the lab to store it in separate, enclosed storage boxes distributed among the different bays of the laboratory. This dry ice would be used by about a dozen people in the synthetic chemistry lab over the course of a couple of days. At the time of the incident, the graduate student was wearing full personal protection equipment, including closed-toe shoes, long pants, a lab coat, nitrile and cryogenic gloves, and safety goggles. Because of the holiday, PS Stores staff members were not on duty, and the graduate student opted to do a self-check-out of the dry ice.

**Figure 1:**
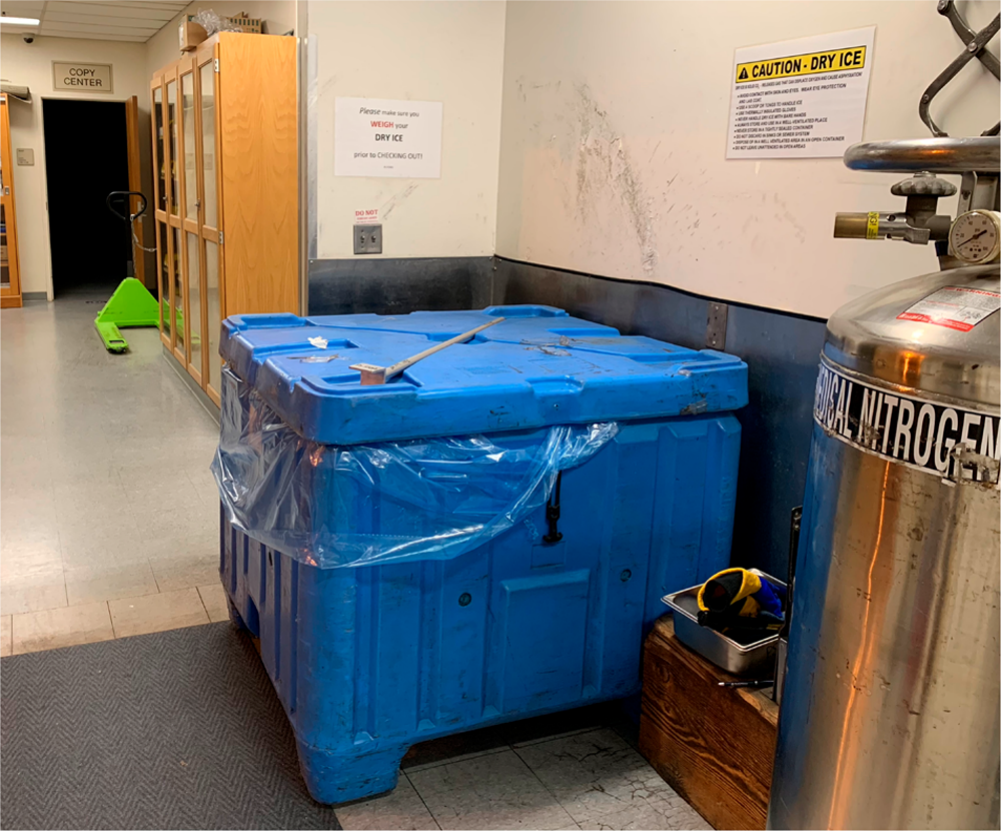
Container used for storage of dry ice at UCI Physical Sciences Stores. The cautionary sign on the wall was introduced after the incident in question. It does not explicitly warn against the action of reaching into the container to retrieve dry ice.

The dry ice packs in the PS Stores are held in a container (Figure 1) with horizontal dimensions of 47″ × 42″ and a depth of 40″ (106.7 cm × 119.4 cm with a 101.6 cm depth), and its upper lip is taller than the student’s waist (student is a healthy individual of medium build and 5′ 5″ (162.5 cm) tall, in their mid-20s). Because PS Stores was running low in supply, the graduate student needed to extend their entire torso inside the container to reach the dry ice at the bottom, and then needed to lift the heavy bags of dry ice.

**Figure 2:**
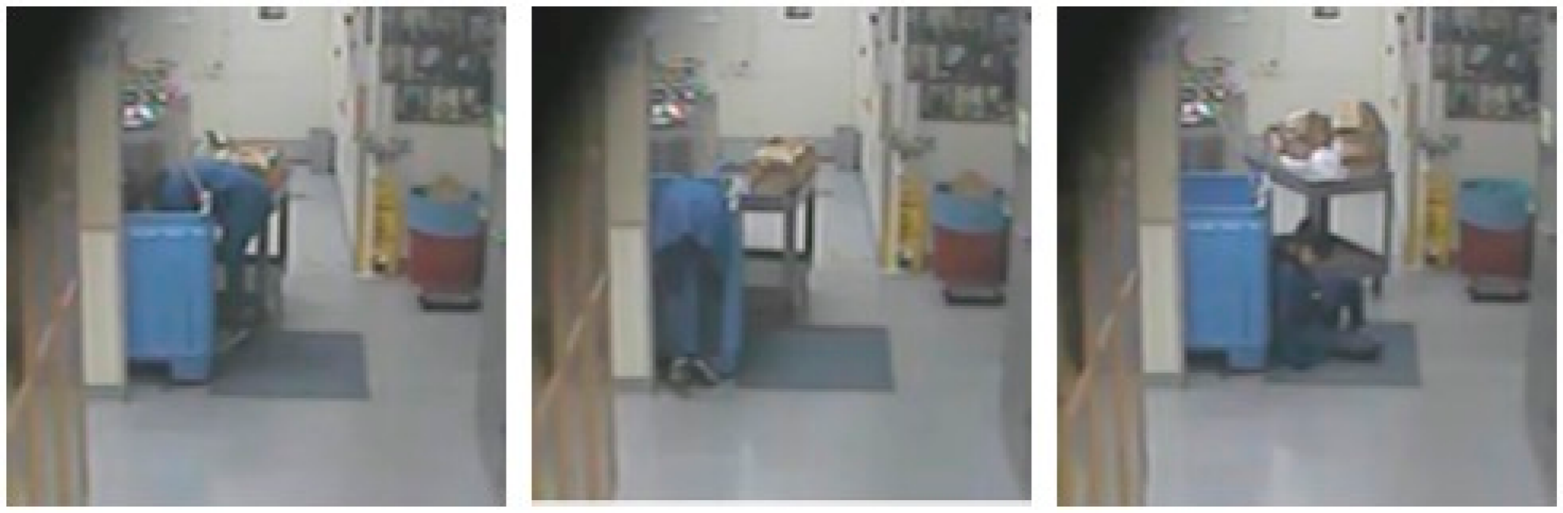
Stills from security camera footage of the incident (approved for release by UCI Public Records Office).

The graduate student repeated this procedure several times, placing ∼180 lb (81.6 kg) of dry ice onto the cart. After placing the final block onto the cart, they became dizzy, had shortness of breath, and lost consciousness (Figure 2). The graduate student fell beside the container in a sitting position, alone. After 10 min, the student woke up, hyperventilating, and called another graduate student in their lab for help. The other graduate student came and assisted the exposed graduate student in leaving the area.

It is evident that, had the person lost consciousness while their torso was extended into the container, the outcome would have been disastrous. Luckily, in this case, the exposed graduate student had a medical check-up, including a chest X-ray and bloodwork, and all results were normal. The student did not experience any burn injuries.

After this safety incident, the exposed student was clinically diagnosed with post-traumatic stress disorder (PTSD). The person continues to have strong negative reactions when encountering situations reminiscent of the incident, such as when entering areas with a cold, positive airflow or handling dry ice for use in cold traps.

## Lessons Learned

### Underlying Dangers

- The container is too deep for a researcher to retrieve dry ice safely when the supplies are nearly exhausted.
- A buddy system was not in place for the job.
- There were no tools provided to expedite the retrieval process.
- There was no oxygen or carbon dioxide oxygen monitoring system, nor enhanced indoor ventilation system.
- There was no Standard Operating Procedure, or warning sign for retrieving dry ice.

### Key Considerations

- Because dry ice, especially in large quantities, is an asphyxiation hazard in a confined space, dry ice storage must be appropriately sized for those working with it to minimize asphyxiation risks.
- A buddy system is critical for prevention of any adverse events from exposure to carbon dioxide and for effective response in the event of any such event.
- A Standard Operating Procedure must be carefully designed with respect to the working environment and tasks. Signs and warnings must be clearly visible.

### Changes in Response to the Incident

After the incident, there were multiple discussions within the Graduate Student Safety Team, faculty advisors, and the environmental health and safety (EH&S) team about how to enable safer retrieval of dry ice for all researchers, with an obvious preference for engineering controls that would diminish or eliminate risk. The following measures were implemented as a result:

- Warning signs and informational posters were posted on the walls near the dry ice container and on bathroom stalls to educate researchers of risks associated with dry ice.
- A large metal clamp tool was provided to help people lift dry ice blocks from the bottom of the container. However, this tool is challenging to use with heavy blocks of dry ice.
- PS Stores implemented a process to regularly monitor the level of dry ice; once below half full, the container is exchanged for a full one such that the dry ice is more easily accessible. The container was also moved away from one wall, affording access to the container from three usable sides.
- Critically, a mandatory buddy system was introduced for when dry ice is retrieved from PS Stores and transported to research laboratories.

We believe that these solutions, in aggregate, significantly reduce the risk associated with retrieval of dry ice blocks from UCI PS Stores. Unfortunately, the Chemistry department, EH&S, and PS Stores staff concluded that it was not plausible to change the size of the container that the supplier uses to provide dry ice, because most bins that are overall smaller in volume are either just as tall or taller than the one currently in use. Dry ice pellets were considered as a possible alternative; however, they would be stored in similar containers and likely require more time within the depths of the container for retrieval.

## Select Previous Relevant Incidents

There are many examples of asphyxiation injuries caused by carbon dioxide released by sublimation of dry ice in enclosed spaces. In 2002, a 51-year-old medical research scientist with a history of mild cold-induced bronchial asthma entered an 8 ft × 8 ft × 14 ft (2.4 m × 2.4 m × 4.3 m) poorly ventilated cold room.^2^ Fifteen 1 L dry ice blocks had been delivered to the room 3 h prior to his entry. About 4 h after last seen alive, the decedent was found on the floor of the cold room. The autopsy report concluded that the cause of death was excessive inhalation of carbon dioxide. A similar incident occurred in 2009, as a 59-year-old freezer technician entered a broken walk-in freezer filled with dry ice.^3^ The autopsy report suggested that the decedent likely sought to exit the freezer room based on the contusions on his right arm and hands and the dents inside the freezer room door. A similar incident occurred in 2021 to a 35-year-old man who worked at a fast-food chain.^4^ He initially experienced acute hypoxic respiratory failure and metabolic acidosis, but was discharged within 24 h subsequent to supplemental oxygen and fluid treatment.

Dry ice asphyxiations in cars have also been reported numerous times.^5^ In 2017, a 62-year-old woman was driving with a container of dry ice and ice cream present with her in the vehicle.^6^ After 15 min of experiencing lightheadedness and shortness of breath, she was found unconscious with her vehicle overturned. Similar asphyxiation events caused deaths in an ice cream truck^7^ and a restaurant delivery truck.^8^

Other examples of asphyxiation injuries by carbon dioxide include the injuries from a fire extinguisher system,^2,9^ transportation of a liquid CO_2_ tank that affected 25 people,^10^ a volcanic explosion,^11^ and onion fermentations.^12^ Most deaths occurred in confined indoor spaces.

## Physiological Effects of CO_2_ Inhalation

Carbon dioxide gas is a normal byproduct of the human respiratory system. But when excess CO_2_ is present in the arterial bloodstream, the “Bohr effect” occurs, where an elevated level of CO_2_ causes hemoglobin to have a lower affinity for O_2_.^13^ The increased partial pressure of CO_2_ in alveoli leads to reaction with water to generate carbonic acid, which triggers respiratory acidosis. Thus, inhalation of significant quantities of CO_2_ lowers the blood pH and impacts cardiological, respiratory, and nervous systems.^12^ Common symptoms of CO_2_ exposure at lower concentrations are hearing loss, tachycardia, hypertension, hyperventilation, headache, and dizziness.^14^ At higher concentrations, convulsion, loss of consciousness, and death have also been reported. Langford published an excellent in-depth review on the mechanism and toxicokinetic and clinical features of CO_2_ poisoning.^14^

## Long-Term Effects of This Exposure

As noted above, the graduate student involved in the incident was deemed physically healthy, but was diagnosed with PTSD and has lingering mental health problems nearly a year after the incident. While preparing this manuscript, we noticed the lack of literature reports on the mental health of researchers after experiencing safety incidents. We would like to encourage those involved in lab safety incidents to share holistic stories of how such events can impact researchers’ lives.

## Conclusion

While it is an excellent coolant for cryogenic baths and serves as a valuable C_1_ source in organic reactions, dry ice must, like all chemicals, be handled carefully and with adequate personal protection equipment and safety measures. This report was disclosed in the hope of preventing dangerous situations related to retrieving dry ice from large containers in laboratory settings. Indeed, in an informal poll by the corresponding author of a number of chemistry faculty members at representative research active institutions, the same dangerous situation existed in many cases. In some instances, direct delivery of smaller quantities of dry ice to laboratories was available; even so, these institutions almost surely have a centralized stockroom or store from which dry ice can be retrieved by less regular users, likely from containers of the sort involved in the incident we describe. We worry that with such a routinely used substance as dry ice, researchers might become desensitized to its potential dangers, particularly with respect to asphyxiation.
